# Liver Fat Changes in Patients With Type 2 Diabetes by Presence of Genetic Hepatic Steatosis: A Post Hoc Analysis of SURPASS‐3 MRI

**DOI:** 10.1111/liv.70837

**Published:** 2026-08-11

**Authors:** Kenneth Cusi, Amalia Gastaldelli, Laura Fernández Landó, Palash Sharma, Amelia Torcello‐Gómez, Ángel Rodríguez

**Affiliations:** ^1^ Division of Endocrinology, Diabetes and Metabolism The University of Florida Gainesville Florida USA; ^2^ Institute of Clinical Physiology, CNR Pisa Italy; ^3^ Eli Lilly and Company Indianapolis Indiana USA; ^4^ Eli Lilly and Company Cork Ireland; ^5^ Lilly Spain Madrid Spain

**Keywords:** fatty liver, human PNPLA3 protein, magnetic resonance imaging, single nucleotide polymorphism, tirzepatide, type 2 diabetes mellitus

## Abstract

The patatin‐like phospholipase 3 (PNPLA3) I148M genetic variant is highly associated with steatotic liver disease. In the SURPASS‐3 MRI substudy, tirzepatide significantly reduced liver fat content (LFC) versus insulin degludec in insulin‐naïve patients with type 2 diabetes with metabolic dysfunction‐associated steatotic liver disease. The influence of PNPLA3 I148M allele on changes in LFC in response to tirzepatide is unknown. This post hoc analysis evaluated changes in MRI‐assessed LFC and cardiometabolic parameters by presence (genotypes GG and CG) or absence (genotype CC) of the PNPLA3 I148M allele after 52‐week treatment. Tirzepatide‐treated participants experienced significant reductions in LFC, weight, HbA_1c_, and overall greater improvement in lipids and liver enzymes regardless of the presence of PNPLA3 I148M allele. Findings were consistent across genotype subgroups. In this exploratory analysis, the presence of PNPLA3 I148M allele did not seem to affect tirzepatide‐induced improvement in LFC and several cardiometabolic parameters in patients with type 2 diabetes.

**Trial Registration:** ClinicalTrials.gov identifier: NCT03882970

## Key Points

- Tirzepatide significantly reduced liver fat compared to insulin degludec in patients with type 2 diabetes and metabolic dysfunction‐associated steatotic liver disease in the SURPASS‐3 MRI substudy.
- The patatin‐like phospholipase 3 (PNPLA3) I148M genetic variant is strongly associated with steatotic liver disease, but whether it affects liver fat changes in response to tirzepatide was unknown.
- This post hoc analysis compared changes in liver fat and cardiometabolic measures in patients with and without the PNPLA3 I148M variant after 52 weeks of treatment.
- The PNPLA3 I148M variant did not appear to affect improvements in liver fat content and cardiometabolic measures observed following tirzepatide treatment in patients with type 2 diabetes.

**Abbreviations**
C: cytosine
G: guanine
HbA_1c_
: glycated haemoglobin
I148M: change in codon 148 from isoleucine to methionine
LFC: liver fat content
MASH: metabolic dysfunction‐associated steatohepatitis
MRI: magnetic resonance imaging
PNPLA3: patatin‐like phospholipase domain‐containing 3
SGLT‐2i: sodium‐glucose co‐transporter‐2 inhibitor
T2D: type 2 diabetes

## 1 Introduction

Steatotic liver disease is associated with type 2 diabetes (T2D) and obesity, among other metabolic complications [1]. The prevalence of steatotic liver disease in people with T2D is ~70%, with ~15%–38% having metabolic dysfunction‐associated steatohepatitis (MASH) with clinically significant fibrosis, prompting the American Diabetes Association to encourage screening of all individuals in this population [2]. Accordingly, current guidelines on the treatment of T2D recommend weight management as part of a multifactorial approach to prevent various complications, also for individuals with T2D and steatotic liver disease at high risk of fibrosis [3, 4].

Genetic factors also play a key role in the development of steatotic liver disease. The genetic variant rs738409 in the patatin‐like phospholipase domain‐containing 3 (PNPLA3) is highly associated with steatotic liver disease [5]. The variant is a cytosine (C) to guanine (G) substitution that changes codon 148 from isoleucine to methionine (I148M) in the PNPLA3 gene.

In the SURPASS‐3 trial, once‐weekly tirzepatide showed greater reductions in glycated haemoglobin (HbA_1c_) and body weight in adults with T2D after 52 weeks of treatment compared to titrated once‐daily insulin degludec [6]. Tirzepatide was also associated with improvements in waist circumference, lipids profile, and blood pressure. In the SURPASS‐3 MRI (magnetic resonance imaging) substudy, tirzepatide showed significant reductions in proton density fat fraction‐derived liver fat content (henceforth LFC) compared with insulin degludec in a subpopulation of patients with high risk of hepatic steatosis in the SURPASS‐3 trial [7]. To date, the influence of the PNPLA3 I148M allele on changes in liver fat in response to tirzepatide is not known.

The aim of this post hoc analysis of the SURPASS‐3 MRI substudy is to evaluate changes in LFC and cardiometabolic parameters by the presence (genotypes GG and CG) or absence (genotype CC) of the PNPLA3 I148M allele.

## 2 Methods

### 2.1 Study Design, Procedures, and Participants

This is a substudy of the phase 3, open‐label SURPASS‐3 trial. The designs and procedures of the main study and substudy have previously been published [6, 7]. Participants were randomized (1:1:1:1) into the main study to once‐weekly tirzepatide 5, 10, or 15 mg or once‐daily insulin degludec. Participants underwent an MRI scan prior to randomisation and at Week 52 for assessment of LFC. Further details on the procedures are in the Data S1.

Participants of the main study were also eligible for this substudy if additional criteria were met. The inclusion and exclusion criteria of the main study and substudy have previously been published [6, 7]. Briefly, participants were insulin‐naive adults with T2D inadequately controlled on metformin with or without a sodium‐glucose co‐transporter‐2 inhibitor (SGLT‐2i). Additional exclusion criteria for this substudy were a fatty liver index < 60, contraindications for MRI scanning, claustrophobia precluding completion of an MRI examination, history of excessive alcohol intake (> 21 units per week for males; > 14 units per week for females), a BMI > 45 kg/m^2^, and participation in the SURPASS‐3 CGM (Continuous Glucose Monitoring) substudy [8].

### 2.2 Outcomes

Genotyping of participant blood DNA was performed using a TaqMan allelic discrimination assay (see Data S1).

The effect of tirzepatide (pooled doses) and insulin degludec at Week 52 was compared for the changes from baseline in LFC, body weight, waist circumference, HbA_1c_, fasting serum glucose, lipids, and liver enzymes by genotype subgroups (CC, CG, and GG).

### 2.3 Statistical Analysis

This post hoc analysis included participants who were enrolled in the SURPASS‐3 MRI substudy, received at least one dose of the study drug, and had a valid MRI scan at either baseline or postbaseline.

Postbaseline MRI performed at any non‐scheduled visit or at an early‐termination visit (if available) was carried forward to the endpoint visit (Week 52). Data collected more than 14 days (MRI data) or more than 7 days (non‐MRI data) after study drug was discontinued or rescue therapy was initiated were excluded from the analysis.

Analysis of covariance was used to analyse MRI data, for which only baseline and Week 52 assessments were conducted. The percentage of participants achieving LFC thresholds was analysed with logistic regression. A mixed model repeated measures model was used for all other outcomes. Fasting lipids and liver enzymes were analysed on log‐transformed data and then converted back to the original scale. All models were adjusted for baseline values and stratification factors (pooled country, baseline oral antihyperglycaemic medication use [metformin or metformin plus an SGLT‐2i], and baseline HbA_1c_ [≤ 8.5% or > 8.5%; ≤ 69 or > 69 mmol/mol]). Between‐genotype comparisons were performed for each treatment arm separately. The treatment‐by‐genotype subgroup interaction effect was also tested. Since this is a post hoc analysis, no multiplicity adjustment was performed.

## 3 Results

### 3.1 Participants

A total of 276 participants were included in this analysis. The percentage of participants of Hispanic or Latino ethnicity was greater in the GG and CG genotype subgroups (Table 1). Differences were observed in sex and the use of concomitant metformin with or without an SGLT‐2i by genotype subgroup. LFC, triglycerides, and very low‐density lipoprotein cholesterol were also statistically different across genotype subgroups (*p* < 0.05), with LFC being greater in the GG and CG genotype subgroups. Other baseline characteristics were similar across genotype subgroups. The demographics and baseline clinical characteristics by treatment arms and genotype subgroup are presented in Table S1.

**TABLE 1:** Demographics and baseline clinical characteristics in the overall population by genotype subgroup.

Population	Overall			*p*
Genotype	CC	CG	GG	
N	120	109	47	
Age, years	57.2 (9.3)	55.5 (10.3)	55.6 (9.9)	0.379
Female, *n* (%)	42 (35.0)	60 (55.0)	16 (34.0)	0.004
Race, *n* (%)				0.634
American Indian or Alaska Native	1 (0.8)	2 (1.8)	0	—
Black or African American	2 (1.7)	0	1 (2.1)	—
Multiple	0	1 (0.9)	0	—
White	117 (97.5)	106 (97.2)	46 (97.9)	—
Ethnicity, *n* (%)				< 0.001
Hispanic or Latino	33 (27.5)	68 (62.4)	35 (74.5)	—
Not Hispanic or Latino	84 (70.0)	40 (36.7)	11 (23.4)	—
Not reported	3 (2.5)	1 (0.9)	1 (2.1)	—
Duration of diabetes, years	8.0 (5.5)	9.2 (8.2)	7.3 (5.5)	0.177
Diabetes medication^a^, *n* (%)				< 0.001
Metformin alone	69 (57.5)	84 (77.1)	40 (85.1)	—
Metformin + SGLT‐2i	51 (42.5)	25 (22.9)	7 (14.9)	—
Blood pressure, mmHg				
Systolic	133.2 (12.3)	131.8 (13.2)	130.0 (14.3)	0.360
Diastolic	80.3 (8.7)	79.4 (9.2)	79.1 (7.2)	0.616
Weight‐related characteristics and liver fat content				
Body mass index, kg/m^2^	33.4 (4.8)	33.5 (4.7)	33.5 (5.4)	0.996
Body weight, kg	96.0 (16.3)	93.0 (17.4)	92.1 (15.0)	0.242
Waist circumference, cm	111.3 (11.5)	110.9 (11.6)	109.1 (12.0)	0.529
Liver fat content, %	14.05 (8.24)	17.56 (9.67)	15.71 (8.31)	0.016
Glucose metabolism				
HbA_1c_, %	8.20 (0.83)	8.41 (1.02)	8.19 (0.91)	0.155
HbA_1c_, mmol/mol	66.1 (9.0)	68.5 (11.2)	66.0 (10.0)	0.155
Fasting serum glucose, mmol/L	9.81 (2.68)	9.85 (2.60)	10.14 (3.04)	0.764
Fasting serum glucose, mg/dL	176.7 (48.3)	177.5 (46.8)	182.7 (54.8)	0.764
Fasting lipids, mg/dL				
Triglycerides	215.3 (135.4)	221.1 (176.6)	158.1 (87.6)	0.039
HDL‐C	43.3 (11.9)	44.2 (10.7)	46.2 (10.0)	0.304
LDL‐C	94.5 (33.3)	103.2 (36.7)	98.8 (31.9)	0.161
VLDL‐C	39.3 (17.5)	40.2 (20.5)	30.8 (15.7)	0.010
Liver enzymes, U/L				
Alanine aminotransferase	28.2 (14.4)	30.5 (18.2)	31.5 (13.8)	0.375
Aspartate aminotransferase	20.8 (10.3)	23.0 (12.0)	22.1 (7.8)	0.313
Gamma glutamyl‐transpeptidase	41.5 (30.7)	40.8 (27.7)	39.3 (44.6)	0.928

*Note:* Genotype CC indicates the absence of the patatin‐like phospholipase 3 (PNPLA3) I148M allele, and genotypes GG and CG indicate the presence of the PNPLA3 I148M allele. Data are mean (SD) or *n* (%).

Abbreviations: HbA_1c_, glycated haemoglobin A_1c_; HDL‐C, high‐density lipoprotein cholesterol; LDL‐C, low‐density lipoprotein cholesterol; SGLT‐2i, sodium‐glucose co‐transporter‐2 inhibitor; VLDL‐C, very low‐density lipoprotein cholesterol.

^a^ Metformin doses ≥ 1500 mg/day.

### 3.2 Changes From Baseline at Week 52

**FIGURE 1:**
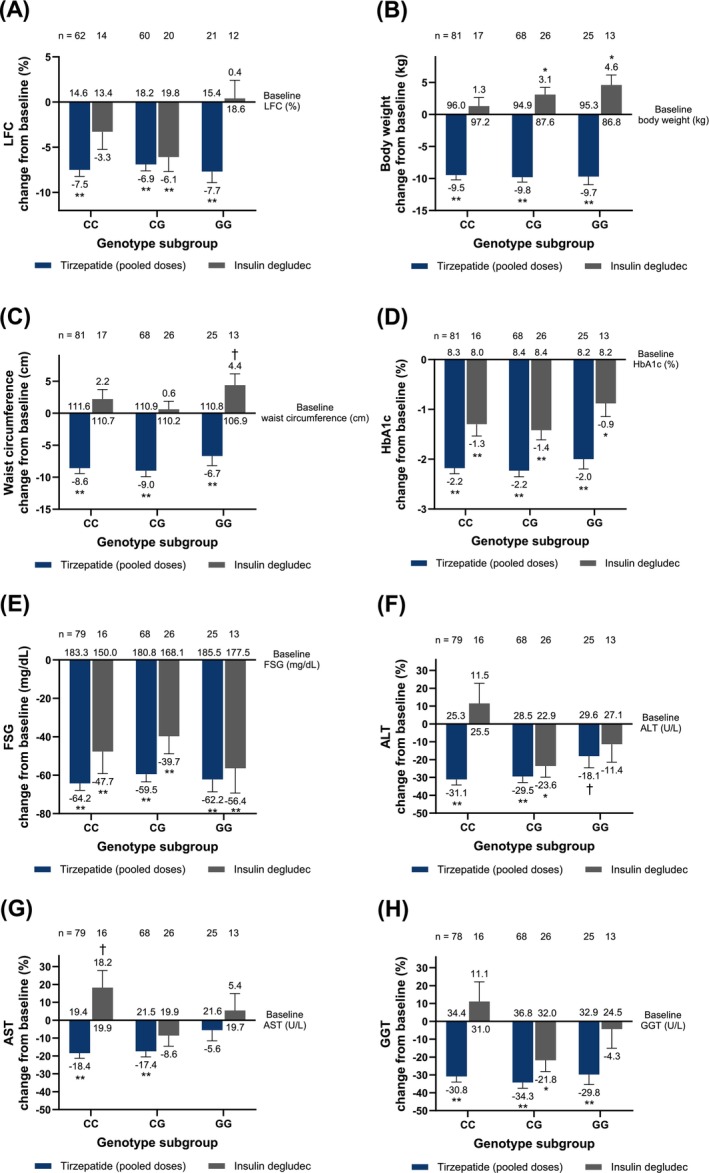
Changes from baseline at Week 52 in (A) LFC, (B) body weight, (C) waist circumference, (D) HbA_1c_, (E) FSG, (F) ALT, (G) AST, and (H) GGT by genotype subgroup. Genotype CC indicates the absence of the patatin‐like phospholipase 3 (PNPLA3) I148M allele, and genotypes GG and CG indicate the presence of the PNPLA3 I148M allele. Data are least‐squares mean and error bars are standard error. †*p* < 0.05, *p < 0.01, and **p < 0.001 versus baseline. Treatment‐by‐genotype subgroup interaction at Week 52 was not significant except for ALT and LFC, which was significant (*p* < 0.05) due to statistical differences between GG and CG genotypes in the insulin degludec arm (*p* < 0.05). ALT, alanine aminotransferase; AST, aspartate aminotransferase; FSG, fasting serum glucose; GGT, gamma glutamyl‐transpeptidase; HbA_1c_, glycated haemoglobin A1c; LFC, liver fat content.

Tirzepatide‐treated participants experienced significant (*p* < 0.001) reductions at Week 52 in LFC, body weight, waist circumference, HbA_1c_, and fasting serum glucose, and overall improvement in lipids profile and liver enzymes regardless of the genotype subgroup (Figure 1 and Table S2). There were no statistically significant differences in any of the reported outcomes between any of the genotype subgroups (CC vs. CG, CC vs. GG, and CG vs. GG). Approximately 30%–40% of tirzepatide‐treated participants with baseline LFC ≥ 6% achieved LFC < 6% at Week 52 regardless of the presence of the G allele (Table S3).

Overall, insulin degludec‐treated participants experienced increases in body weight and waist circumference and significant decreases (*p* < 0.01) in HbA_1c_ and fasting serum glucose regardless of the genotype subgroup. There were no statistically significant differences in any of the reported outcomes between genotype subgroups except for LFC between CG and GG genotypes (*p* < 0.05) (Figure 1A). However, changes in LFC were not statistically different between the G‐allele carriers and the CC subgroup (CC vs. CG and CC vs. GG). There were also statistically significant differences between the CC and CG genotypes for alanine aminotransferase, aspartate aminotransferase, and gamma‐glutamyl transpeptidase (Figure 1F–H, respectively).

## 4 Discussion

To our knowledge, this is the first analysis assessing the influence of the PNPLA3 I148M allele (genotypes GG and CG versus CC) on changes in liver fat in response to tirzepatide in people with T2D. Reductions in LFC and improvements in cardiometabolic parameters at Week 52 were consistent across genotype subgroups in tirzepatide‐treated participants and in line with the primary results of the SURPASS‐3 MRI substudy [7]. Thus, there seems to be no influence of the presence of the PNPLA3 I148M allele on the therapeutic efficacy of tirzepatide in this population. Interestingly, participants in the insulin degludec group seemed to behave differently, and while not statistically significant overall, the genetic background may have had a negative impact on the ability to ameliorate LFC or HbA_1c_, as well as on body weight and waist circumference. There was not a clear trend in the statistical differences between genotype subgroups for liver enzymes with insulin degludec, although interpretation is limited by the small sample size. Nevertheless, prior analyses pooling G‐allele carriers demonstrated consistent tirzepatide benefit regardless of PNPLA3 I148M allele status [9] (Figure S1), supporting a robust treatment effect across genotypes.

It has been reported that the GG genotype confers a higher risk of steatotic liver disease, but these patients are more sensitive to the beneficial effects of lifestyle modification [10, 11]. However, there is limited and conflicting evidence regarding the influence of the GG genotype on the beneficial effects of different types of antihyperglycaemic medication. Two small studies in patients with T2D and steatotic liver disease suggested that the beneficial effects of alogliptin on liver function and of the combination of dapagliflozin and n‐3 carboxylic acids on LFC were greater in patients with GG and CG genotypes [12, 13]. Conversely, treatment with dapagliflozin alone or exenatide improved LFC in the CC genotype subgroup better than in the GG + CG genotype subgroup in patients with T2D [13, 14]. Nevertheless, there is a lack of replication studies with antihyperglycaemic medications. Whether these observations may further translate into histological endpoints benefits is still uncertain. Recent preliminary data from the SYNERGY‐NASH study (NCT04166773) in people with MASH suggest that the G‐risk allele may be related to MASH independently of its effect on liver fat [15].

This post hoc analysis had limited power for genotype subgroup comparisons due to small sample sizes (GG insulin degludec arm, particularly), increasing Type II error risk. Larger studies are needed to evaluate genotype‐treatment interactions and the relative contribution of different factors to LFC changes by genotype, as baseline LFC and body weight change account for only 50% of variation in LFC reduction in the overall study population [7]. Indeed, these analyses were not adjusted for baseline body weight or ethnicity, which may confound observed outcomes.

Further limitations include the small non‐White population and imbalance in the percentage of participants of Hispanic or Latino ethnicity across genotype subgroups. As some genotype differences between ethnic groups have been described [5], further studies are needed to understand whether the findings from this analysis are generalizable to different racial/ethnic groups. The unavailability of postbaseline MRI measures for some participants or liver biopsy results is an additional limitation of this work.

In this post hoc analysis in people with T2D from the SURPASS‐3 MRI substudy, the presence of the PNPLA3 I148M allele did not seem to have a significant influence on the effect of tirzepatide on LFC and several cardiometabolic parameters.

## Author Contributions

Á.R. and L.F.L. contributed to the substudy design. Á.R. provided medical oversight during the trial. Á.R. and A.T.‐G. drafted the manuscript. K.C. and A.G. contributed to the medical interpretation of the data. P.S. was responsible for the statistical analyses. Á.R. and P.S. verified the data and are the guarantors of this work and, as such, take responsibility for the integrity of the data and the accuracy of the data analysis. All authors participated in the interpretation of the data and critical review of the manuscript, had full access to all the data in the substudy, and approved of this manuscript to be submitted for publication.

## Funding

This study was funded by Eli Lilly and Company.

## Ethics Statement

The substudy protocol was approved by the Ethical Review Board at each site and was followed according to local regulations and the principles of the Declaration of Helsinki, Council of International Organizations of Medical Sciences International Ethical Guidelines and Good Clinical Practice guidelines.

## Consent

Written informed consent to participate in this specific substudy was obtained from all participants at the screening visit.

## Conflicts of Interest

K.C. has received research support towards the University of Florida as principal investigator from Boehringer Ingelheim, Echosens, Inventiva, LabCorp and Perspectum; has served as a consultant for Arrowhead, 89Bio, Boehringer Ingelheim, Bristol Myers Squibb, Dexcom, Echosens, Eli Lilly and Company, GSK, MGGM, Novo Nordisk, Sagimet Biosciences, Terns Pharmaceuticals and Zealand Pharma. A.G. has served as a consultant/on advisory boards for Boehringer Ingelheim, Madrigal, Merck Sharp & Dohme, Novo Nordisk and Regeneron; and has received speaker's honoraria, travel support or other educational fees from Boehringer Ingelheim, Merck Sharp & Dohme, Novo Nordisk, Madrigal and Mercodia. L.F.L., P.S., A.T.‐G. and Á.R. are employees and shareholders of Eli Lilly and Company.

## Supporting information


**Data S1:** liv70837‐sup‐0001‐Supinfo.docx.
**Figure S1:** Changes from baseline at Week 52 in (A) LFC, (B) body weight, (C) waist circumference, (D) HbA_1c_, (E) ALT, (F) AST, aβnd (G) GGT by presence of patatin‐like phospholipase 3 (PNPLA3) I148M allele (yes [genotype CG/GG] or no [genotype CC]). Data are least‐squares mean and error bars are standard error. ^#^ *p* = 0.093, *p < 0.05, and **p ≤ 0.001 versus insulin degludec. Treatment‐by‐genotype subgroup interaction at Week 52 was not significant. ALT = alanine aminotransferase. AST = aspartate aminotransferase. GGT = gamma glutamyl‐transpeptidase. HbA_1c_ = glycated haemoglobin A1c. LFC = liver fat content.
**Table S1:** Demographics and baseline clinical characteristics in the tirzepatide and insulin degludec arms by genotype subgroup.
**Table S2:** Changes in fasting lipids at Week 52 by genotype subgroup.
**Table S3:** Percentage of participants achieving liver fat content < 6% at Week 52 by genotype subgroup.

## Data Availability

Eli Lilly and Company provides access to all individual participant data collected during the trial, after anonymisation, with the exception of pharmacokinetic or genetic data. Data are available to request 6 months after the indication studied has been approved in the US and EU and after primary publication acceptance, whichever is later. No expiration date of data requests is currently set once data are made available. Access is provided after a proposal has been approved by an independent review committee identified for this purpose and after receipt of a signed data sharing agreement. Data and documents, including the study protocol, statistical analysis plan, and blank or annotated case report forms, will be provided in a secure data sharing environment. For details on submitting a request, see the instructions provided at www.vivli.org.
