# Supplementary material for: Liver Fat Changes in Patients With Type 2 Diabetes by Presence of Genetic Hepatic Steatosis: A Post Hoc Analysis of SURPASS‐3 MRI
Source: Liver Int. 2026 Aug 11;46(9):e70837. doi: 10.1111/liv.70837 (PMC13458421; doi:10.1111/liv.70837)
Supplement: Supplementary file 1 [file LIV-46-0-s001.docx]

**SUPPORTING INFORMATION**

**Procedures**

In addition to the main trial procedures, during the one-week screening period, all eligibility requirements specific to this substudy were assessed in the participants invited to be part of the MRI substudy. Just after the screening period and two weeks before starting the main trial and the substudy, fasting triglycerides were measured to determine the fatty liver index, in order to assess the participant’s eligibility to enter the MRI substudy. All eligible participants underwent an MRI scan during the two-week lead-in period to determine the baseline liver fat content (LFC). At Week 0, eligible participants underwent blood collection, prior to the randomisation procedures, to determine other baseline laboratory measures. After 52 weeks of treatment or early discontinuation (if feasible), postbaseline MRI scan and blood collection were performed (within ±7 days of the date of the main study visit for MRI scan).

Blood samples were collected from participants after a fast of at least eight hours without having taken any doses of their study drug and concomitant metformin with or without a sodium-glucose co-transporter-2 inhibitor.

**Study drug**

The starting dose of tirzepatide was 2.5 mg for four weeks, then the dose was increased 2.5 mg every four weeks until the randomised dose was achieved. The starting dose of insulin degludec was 10 U/day, titrated weekly to a fasting blood glucose of <5.0 mmol/L (<90 mg/dL), following a treat-to-target (TTT) algorithm based on the median value of the last three pre-breakfast self-monitored blood glucose values. Insulin adjustments different than the TTT algorithm could be decided as per the investigator’s discretion in case of safety concerns.

**Magnetic resonance imaging**

MRI scans were performed after an overnight fast of at least eight hours at 1.5 T or 3 T at each site following a standardised imaging acquisition protocol. The same scanner (Siemens, Munich, Germany; Philips, Amsterdam, Netherlands; or General Electric, Chicago, IL, USA) and imaging acquisition parameters were used for both baseline and postbaseline time points for each participant. LFC was assessed with MRI-proton density fat fraction (PDFF) and was expressed as the mean fat fraction across all nine user-defined regions of interest in the liver. The average value of the nine regions of interest was analysed as the mean LFC. The regions of interest were defined on the shortest time to echo image of each slice. MRI scans were evaluated in the imaging core laboratory for independent central review by a qualified vendor (BioTel™ Research; Cardiocore and VirtualScopics; Rochester, NY, USA).

**PNPLA3 I148M genotyping**

DNA was extracted from whole EDTA-treated blood using the MagNA Pure 96 DNA and Viral NA Small Volume Kit (Roche). Then, the DNA sample was used in a reaction mix containing the TaqPath™ ProAmp™ Master Mix (ThermoFisher Scientific) and the TaqMan™ SNP (single nucleotide polymorphism) Genotyping Assay (ThermoFisher Scientific). The reaction mix was loaded into a LightCycler® 480 (Roche) to determine the allelic content of the sample. The PNPLA3 I148M genotyping assay was a multiplexed, endpoint, TaqMan allelic discrimination assay. The assay included one set of gene-specific primers paired with two allele-specific probes to detect the two variants of a SNP site in the targeted PNPLA3 gene sequence.

**Statistical analysis**

For the pooled analysis (Figure S1), changes from baseline to Week 52 in LFC and cardiometabolic parameters were evaluated by presence or absence of PNPLA3 I148M allele (yes [genotype CG/GG] or no [genotype CC]). For assessment of LFC using MRI data, analysis of covariance was used, for which only baseline and Week 52 assessments were conducted. Mixed model repeated measures model was used for all other outcomes. Liver enzymes were analysed on log-transformed data. All models were adjusted for baseline values and stratification factors (pooled country, baseline oral antihyperglycaemic medication use [metformin or metformin plus an SGLT-2i], and baseline HbA_1c_ [≤8.5% or >8.5%; ≤69 or >69 mmol/mol]). Between-treatment comparisons were performed for each genotype subgroup separately. The treatment-by-PNPLA3 I148M subgroup interaction effect was also tested. Since this is a post hoc analysis, no multiplicity adjustment was performed.

**FIGURE S1** Changes from baseline at Week 52 in (A) LFC, (B) body weight, (C) waist circumference, (D) HbA_1c_, (E) ALT, (F) AST, and (G) GGT by presence of patatin-like phospholipase 3 (PNPLA3) I148M allele (yes [genotype CG/GG] or no [genotype CC]). Data are least-squares mean and error bars are standard error. ^#^p=0.093, *p<0.05, and **p≤0.001 versus insulin degludec. Treatment-by-genotype subgroup interaction at Week 52 was not significant. ALT=alanine aminotransferase. AST=aspartate aminotransferase. GGT= gamma glutamyl-transpeptidase. HbA_1c_=glycated haemoglobin A1c. LFC=liver fat content.

**TABLE S1** Demographics and baseline clinical characteristics in the tirzepatide and insulin degludec arms by genotype subgroup

| **Treatment arm** | **Tirzepatide (pooled doses)** | | | **Insulin degludec** | | |
| --- | --- | --- | --- | --- | --- | --- |
| **Genotype** | **CC** | **CG** | **GG** | **CC** | **CG** | **GG** |
| **N** | **96** | **80** | **31** | **24** | **29** | **16** |
| Age, years | 56.7 (9.2) | 55.7 (10.3) | 56.2 (10.7) | 59.1 (9.4) | 55.2 (10.4) | 54.4 (8.1) |
| Female, n (%) | 34 (35.4) | 42 (52.5) | 11 (35.5) | 8 (33.3) | 18 (62.1) | 5 (31.3) |
| Race, n (%) |  |  |  |  |  |  |
| American Indian or Alaska Native | 0 | 2 (2.5) | 0 | 1 (4.2) | 0 | 0 |
| Black or African American | 2 (2.1) | 0 | 1 (3.2) | 0 | 0 | 0 |
| Multiple | 0 | 1 (1.3) | 0 | 0 | 0 | 0 |
| White | 94 (97.9) | 77 (96.3) | 30 (96.8) | 23 (95.8) | 29 (100.0) | 16 (100.0) |
| Ethnicity, n (%) |  |  |  |  |  |  |
| Hispanic or Latino | 23 (24.0) | 49 (61.3) | 23 (74.2) | 10 (41.7) | 19 (65.5) | 12 (75.0) |
| Not Hispanic or Latino | 70 (72.9) | 30 (37.5) | 7 (22.6) | 14 (58.3) | 10 (34.5) | 4 (25.0) |
| Not reported | 3 (3.1) | 1 (1.3) | 1 (3.2) | 0 | 0 | 0 |
| Duration of diabetes, years | 8.3 (5.6) | 9.6 (9.0) | 8.2 (6.2) | 6.8 (4.7) | 8.1 (5.4) | 5.4 (3.5) |
| Diabetes medication^a^, n (%) |  |  |  |  |  |  |
| Metformin alone | 51 (53.1) | 64 (80.0) | 25 (80.6) | 18 (75.0) | 20 (69.0) | 15 (93.8) |
| Metformin + SGLT-2i | 45 (46.9) | 16 (20.0) | 6 (19.4) | 6 (25.0) | 9 (31.0) | 1 (6.3) |
| Blood pressure, mmHg |  |  |  |  |  |  |
| Systolic | 132.4 (12.7) | 131.9 (13.1) | 129.2 (14.0) | 136.4 (10.3) | 131.7 (13.8) | 131.7 (15.2) |
| Diastolic | 80.6 (9.0) | 78.9 (8.2) | 78.5 (7.4) | 79.4 (7.6) | 80.8 (11.4) | 80.3 (7.0) |
| Weight-related characteristics and liver fat content |  |  |  |  |  |  |
| Body mass index, kg/m^2^ | 33.4 (4.9) | 33.7 (4.4) | 34.4 (5.5) | 33.4 (4.6) | 32.8 (5.3) | 31.8 (4.7) |
| Body weight, kg | 95.8 (16.7) | 94.8 (16.6) | 95.3 (14.9) | 97.2 (15.0) | 88.0 (18.9) | 85.9 (13.6) |
| Waist circumference, cm | 111.5 (11.1) | 111.1 (11.7) | 110.8 (11.0) | 110.7 (13.2) | 110.6 (11.3) | 105.9 (13.4) |
| Liver fat content, % | 14.27 (8.51) | 16.82 (8.97) | 15.08 (8.13) | 13.26 (7.31) | 19.55 (11.24) | 16.97 (8.83) |
| Glucose metabolism |  |  |  |  |  |  |
| HbA_1c_, % | 8.25 (0.85) | 8.42 (1.00) | 8.23 (0.75) | 7.96 (0.72) | 8.40 (1.10) | 8.10 (1.18) |
| HbA_1c_, mmol/mol | 66.7 (9.2) | 68.5 (10.9) | 66.5 (8.2) | 63.5 (7.8) | 68.3 (12.0) | 65.0 (12.9) |
| Fasting serum glucose, mmol/L | 10.18 (2.67) | 10.03 (2.68) | 10.30 (3.18) | 8.33 (2.20) | 9.35 (2.32) | 9.85 (2.83) |
| Fasting serum glucose, mg/dL | 183.3 (48.1) | 180.8 (48.3) | 185.5 (57.3) | 150.0 (39.7) | 168.5 (41.8) | 177.4 (50.9) |
| Fasting lipids, mg/dL |  |  |  |  |  |  |
| Triglycerides | 220.0 (136.9) | 215.5 (168.3) | 141.6 (67.6) | 196.3 (130.3) | 236.5 (200.1) | 190.0 (113.0) |
| HDL-C | 42.6 (12.3) | 43.4 (10.5) | 46.4 (10.5) | 45.9 (10.3) | 46.5 (11.1) | 45.9 (9.1) |
| LDL-C | 95.5 (34.9) | 100.8 (36.7) | 92.2 (30.4) | 90.2 (25.9) | 109.7 (36.7) | 111.7 (31.7) |
| VLDL-C | 39.8 (17.2) | 40.8 (21.0) | 28.3 (13.5) | 37.3 (18.9) | 38.6 (19.3) | 35.6 (18.6) |
| Liver enzymes, U/L |  |  |  |  |  |  |
| Alanine aminotransferase | 28.2 (15.1) | 32.3 (19.3) | 32.9 (14.9) | 27.8 (11.3) | 25.6 (13.7) | 28.6 (11.3) |
| Aspartate aminotransferase | 21.0 (11.2) | 23.7 (13.1) | 22.9 (7.0) | 20.0 (5.9) | 20.9 (8.1) | 20.7 (9.3) |
| Gamma glutamyl-transpeptidase | 43.5 (32.9) | 42.6 (29.5) | 45.0 (52.2) | 33.3 (17.6) | 35.9 (21.7) | 28.2 (21.2) |

Genotype CC indicates the absence of the patatin-like phospholipase 3 (PNPLA3) I148M allele, and genotypes GG and CG indicate the presence of the PNPLA3 I148M allele. Data are mean (SD) or n (%). HbA_1c_=glycated haemoglobin A_1c_. HDL-C=high-density lipoprotein cholesterol. LDL-C=low-density lipoprotein cholesterol. SGLT-2i=sodium-glucose co-transporter-2 inhibitor. VLDL-C=very low-density lipoprotein cholesterol. ^a^Metformin doses ≥1500 mg/day.

**TABLE S2** Changes in fasting lipids at Week 52 by genotype subgroup

| **Treatment arm** | **Tirzepatide (pooled doses)** | | | **Insulin degludec** | | |
| --- | --- | --- | --- | --- | --- | --- |
| **Genotype** | **CC** | **CG** | **GG** | **CC** | **CG** | **GG** |
| **n** | **79** | **68** | **25** | **16** | **26** | **13** |
| **Triglycerides, mg/dL** |  |  |  |  |  |  |
| Baseline | 193.2 (11.2) | 189.2 (11.7) | 133.8 (14.0) | 142.1 (19.3) | 193.8 (21.9) | 166.4 (25.7) |
| Week 52 | 138.9 (6.4) | 147.9 (7.1) | 127.3 (10.3) | 157.4 (16.9) | 158.3 (13.2) | 163.6 (19.3) |
| Percent change from baseline; p value vs baseline | -24.0 (3.5); p<0.001 | -19.2 (3.9); p<0.001 | -30.4 (5.6); p<0.001 | -8.8 (9.8); p=0.394 | -8.3 (7.7); p=0.305 | -5.2 (11.2); p=0.650 |
| Difference vs CC (95% CI); p value vs CC | - | -6.0 (-17.9, 7.5); p=0.362 | 9.2 (-9.7, 31.9); p=0.362 | - | -0.6 (-24.8, 31.4); p=0.966 | -3.8 (-30.5, 33.1); p=0.812 |
| **HDL-C, mg/dL** |  |  |  |  |  |  |
| Baseline | 40.0 (1.1) | 41.5 (1.2) | 44.3 (2.1) | 45.4 (2.4) | 44.7 (2.0) | 45.1 (2.7) |
| Week 52 | 45.7 (0.9) | 44.7 (0.9) | 42.2 (1.5) | 44.0 (1.9) | 43.8 (1.5) | 42.7 (2.0) |
| Percent change from baseline; p value vs baseline | 11.3 (2.2); p<0.001 | 8.7 (2.2);  p<0.001 | 2.7 (3.5);  p=0.438 | -1.5 (4.2); p=0.725 | -1.9 (3.3); p=0.569 | -4.5 (4.5); p=0.336 |
| Difference vs CC (95% CI); p value vs CC | - | 2.4 (-3.4, 8.5); p=0.425 | 8.4 (-0.1, 17.5); p=0.052 | - | 0.4 (-10.2, 12.3); p=0.938 | 3.2 (-9.4, 17.5); p=0.632 |
| **LDL-C, mg/dL** |  |  |  |  |  |  |
| Baseline | 87.8 (4.2) | 94.0 (4.8) | 85.4 (7.4) | 85.6 (6.2) | 107.5 (6.5) | 103.0 (8.4) |
| Week 52 | 85.7 (3.3) | 85.3 (3.4) | 87.3 (5.8) | 85.6 (7.3) | 91.8 (6.1) | 91.1 (8.5) |
| Percent change from baseline; p value vs baseline | -4.8 (3.6); p=0.201 | -5.3 (3.8); p=0.171 | -3.1 (6.5); p=0.636 | -14.2 (7.3); p=0.079 | -8.0 (6.1); p=0.216 | -8.7 (8.5); p=0.332 |
| Difference vs CC (95% CI); p value vs CC | - | 0.6 (-10.2,12.6); p=0.922 | -1.7 (-16.1, 15.0); p=0.827 | - | -6.8 (-25.3, 16.3); p=0.525 | -6.0 (-27.3, 21.5); p=0.628 |
| **VLDL-C, mg/dL** |  |  |  |  |  |  |
| Baseline | 36.5 (1.9) | 37.2 (2.0) | 26.7 (2.5) | 28.6 (3.2) | 35.2 (3.3) | 31.8 (4.1) |
| Week 52 | 28.0 (1.2) | 29.1 (1.3) | 25.5 (2.0) | 30.6 (3.0) | 31.8 (2.4) | 32.9 (3.5) |
| Percent change from baseline; p value vs baseline | -20.9 (3.4); p<0.001 | -17.8 (3.7); p<0.001 | -28.0 (5.5); p<0.001 | -6.4 (9.0); p=0.494 | -2.9 (7.3); p=0.698 | 0.5 (10.6); p=0.960 |
| Difference vs CC (95% CI); p value vs CC | - | -3.8 (-15.4, 9.3); p=0.546 | 9.9 (-8.1, 31.5); p=0.299 | - | -3.6 (-25.0, 23.8); p=0.768 | -6.9 (-30.5, 24.6); p=0.623 |

Genotype CC indicates the absence of the patatin-like phospholipase 3 (PNPLA3) I148M allele, and genotypes GG and CG indicate the presence of the PNPLA3 I148M allele. Data are Estimate (standard error), unless otherwise specified. Treatment-by-genotype subgroup interaction at Week 52 was not significant (p>0.05). CI=confidence interval. HDL-C=high-density lipoprotein cholesterol. LDL-C=low-density lipoprotein cholesterol. VLDL-C=very low-density lipoprotein cholesterol.

**TABLE S3** Percentage of participants achieving liver fat content <6% at Week 52 by genotype subgroup

| **Treatment arm** | **Tirzepatide (pooled doses)** | | | **Insulin degludec** | | |
| --- | --- | --- | --- | --- | --- | --- |
| **Genotype** | **CC** | **CG** | **GG** | **CC** | **CG** | **GG** |
| N | 62 | 60 | 21 | 14 | 20 | 12 |
| Liver fat content <6% | 38.89 (7.54) | 37.61 (7.15) | 39.76 (12.37) | 20.83 (13.24) | 21.56 (12.01) | 11.68 (10.27) |
| Odds ratio vs CC (95% CI); p value vs CC | - | 0.95 (0.39, 2.29); p=0.904 | 1.04 (0.30, 3.58); p=0.954 | - | 1.05 (0.17, 6.48); p=0.962 | 0.50 (0.05, 5.15); p=0.562 |
| N | 52 | 54 | 21 | 11 | 16 | 12 |
| Liver fat content <6% (baseline liver fat content ≥6%) | 31.34 (7.52) | 31.01 (7.03) | 40.50 (12.48) | 23.73 (14.75) | 17.37 (11.00) | 12.89 (10.41) |
| Odds ratio vs CC (95% CI); p value vs CC | - | 0.99 (0.38, 2.53); p=0.975 | 1.49 (0.41, 5.39); p=0.542 | - | 0.68 (0.09, 5.30); p=0.709 | 0.48 (0.05, 4.70); p=0.525 |

Genotype CC indicates the absence of the patatin-like phospholipase 3 (PNPLA3) I148M allele, and genotypes GG and CG indicate the presence of the PNPLA3 I148M allele. Data are least-squares mean (standard error), unless otherwise specified. Treatment-by-genotype subgroup interaction at Week 52 was not significant (p>0.05). CI=confidence interval.
